# Mitogen-activated protein kinases and NFκB are involved in SP-A-enhanced responses of macrophages to mycobacteria

**DOI:** 10.1186/1465-9921-10-60

**Published:** 2009-07-01

**Authors:** Joseph P Lopez, David J Vigerust, Virginia L Shepherd

**Affiliations:** 1 Department of Veterans' Affairs Medical Center, Nashville, TN, USA; 2 Department of Pathology, Vanderbilt University, Nashville, TN, USA; 3 Department of Pediatrics, Vanderbilt University, Nashville, TN, USA

## Abstract

**Background:**

Surfactant protein A (SP-A) is a C-type lectin involved in surfactant homeostasis as well as host defense in the lung. We have recently demonstrated that SP-A enhances the killing of bacillus Calmette-Guerin (BCG) by rat macrophages through a nitric oxide-dependent pathway. In the current study we have investigated the role of tyrosine kinases and the downstream mitogen-activated protein kinase (MAPK) family, and the transcription factor NFκB in mediating the enhanced signaling in response to BCG in the presence of SP-A.

**Methods:**

Human SP-A was prepared from alveolar proteinosis fluid, and primary macrophages were obtained by maturation of cells from whole rat bone marrow. BCG-SP-A complexes were routinely prepared by incubation of a ratio of 20 μg of SP-A to 5 × 10^5 ^BCG for 30 min at 37°C. Cells were incubated with PBS, SP-A, BCG, or SP-A-BCG complexes for the times indicated. BCG killing was assessed using a 3H-uracil incorporation assay. Phosphorylated protein levels, enzyme assays, and secreted mediator assays were conducted using standard immunoblot and biochemical methods as outlined.

**Results:**

Involvement of tyrosine kinases was demonstrated by herbimycin A-mediated inhibition of the SP-A-enhanced nitric oxide production and BCG killing. Following infection of macrophages with BCG, the MAPK family members ERK1 and ERK2 were activated as evidence by increased tyrosine phosphorylation and enzymatic activity, and this activation was enhanced when the BCG were opsonized with SP-A. An inhibitor of upstream kinases required for ERK activation inhibited BCG- and SP-A-BCG-enhanced production of nitric oxide by approximately 35%. Macrophages isolated from transgenic mice expressing a NFκB-responsive luciferase gene showed increased luciferase activity following infection with BCG, and this activity was enhanced two-fold in the presence of SP-A. Finally, lactacystin, an inhibitor of IκB degradation, reduced BCG- and SP-A-BCG-induced nitric oxide production by 60% and 80% respectively.

**Conclusion:**

These results demonstrate that BCG and SP-A-BCG ingestion by macrophages is accompanied by activation of signaling pathways involving the MAP kinase pathway and NFκB.

## Background

It is estimated that one-third of the world's population is infected with *Mycobacterium tuberculosis*, with over three million deaths and eight million new cases per year [1]. The causative agent of this disease is an obligate intra-macrophage pathogen that survives within immature phagosomes of these cells [2]. The success of this organism in causing disease is intimately related to its ability to evade killing by the resident macrophages. Thus, mycobacteria have devised ingenious strategies to evade killing by the very host cell that they depend on for survival [3]. At least two processes have been reported as key to the ability of the ingested bacteria to survive. First, mycobacteria enter macrophages via receptor-mediated processes, move to an immature phagosome stage, and actively block maturation of the phagosome and ultimate fusion with lysosomes [4-7]. Second, mycobacteria subvert signalling pathways that lead to production of potentially lethal mediators [8]. The ability of host factors to overcome these mycobacterial strategies is the focus of the current study.

The initial interaction between the host macrophage and mycobacteria results in the induction of intracellular signalling pathways that connect receptor-mediated events to transcriptional activation in the nucleus. Bacillus Calmette-Guerin (BCG) and other mycobacteria enter macrophages after engaging host cell receptors, and activate a series of pathways during this process. These signals can lead to production of immune effector molecules that are critical for limiting the lifespan of the internalized microbes. However, our understanding of the signalling pathways that are stimulated during mycobacterial infection and how the mycobacteria modulate these pathways is limited. Recent studies suggest that one possible strategy might involve regulation and activation of protein tyrosine kinases (PTKs) [9] that subsequently activate members of the STAT pathway, PI3K/Akt pathway and mitogen-activated protein (MAP) kinase family [10-12]. MAP kinases are a family of serine/threonine kinases that are activated by phosphorylation of conserved tyrosine residues [13]. Multiple members of this family including the p42/p44 extracellular signal-regulated kinases (ERK1/2), c-Jun amino-terminal kinases (JNKs), and p38 MAP kinase have been reported to be involved in inflammatory mediator production in response to a wide variety of microbial stimuli. For example, ERK activation is involved in response to *Salmonella *infection of macrophages [14], and MAP kinase activation is required for tumor necrosis factor-α (TNF) production in response to Group B streptococcus infection [15]. Additionally, a number of laboratories have shown that MAP kinases are involved in macrophage activation following exposure to lipopolysaccharide (LPS) and other bacterial cell wall components [13,16]. Recent studies have begun to investigate the role of these kinases in mycobacterial signalling [17]. Early studies by Chan et al showed that the cell wall component of mycobacteria – lipoarabinomannan (LAM) – stimulated nitric oxide production through a pathway involving ERK and JNK [18]. In addition, a number of studies have shown that infection of macrophages with intact mycobacteria activate specific MAP kinases [8,19,20]. Further supporting a role for the importance of these kinases in controlling microbial infection are the findings that pathogenic strains of various bacteria block inflammatory mediator production through inhibition of MAP kinases [21-23].

Following activation, MAP kinases phosphorylate specific transcription factors leading to modulation of cytokine gene transcription. A key transcription factor involved in the up-regulation of many cytokines and other mediators essential to host defense is nuclear factor (NF)κB [24]. Genes regulated by this factor encode a number of proteins involved in the early response to pathogens. Several groups have recently reported activation of NFκB in response to both intact mycobacteria and mycobacterial cell wall components [18,25-27], and NFκB activation has been reported in monocytes of patients infected with *M. tuberculosis *[28,29].

Our laboratory has been studying the role that host factors play in enhancing the innate response to challenge by invading mycobacteria. One of these factors is surfactant-associated protein A (SP-A), a member of the C-type lectin family that is synthesized and secreted by type II epithelial cells in the lung [30]. Work from a number of laboratories has demonstrated that SP-A plays a major role in the clearance of a variety of respiratory pathogens during the innate host response. *In vitro *studies have shown that SP-A functions as an opsonin and enhances the ingestion of such pathogens as BCG [31], *Mycobacterium tuberculosis *[32], influenza A virus [33],*E. coli *[34], *Haemophilus influenzae *[35], *Staphylococcus aureus *[36], *Streptococcus pneumoniae *[37], *Mycoplasma pulmonis *[38] and *Klebsiella pneumoniae *[39]. The importance of SP-A in *in vivo *host defense has been supported recently by the demonstration that mice deficient in SP-A show decreased resistance to group B streptococcal and *Pseudomonas aeruginosa *pneumonia [40,41], decreased clearance of respiratory syncytial virus [42], and reduced killing of mycoplasma [43]. In *in vitro *studies, Kabha et al. and Hickman-Davis et al. demonstrated that SP-A enhances the ingestion and killing of *K. pneumoniae *[39] and mycoplasma [38] by macrophages.

Recent work from our laboratory has shown that SP-A enhances clearance of BCG and avirulent *Mycobacterium tuberculosis *(H37Ra) by cultured rat macrophages [44]. This enhanced clearance is accompanied by increased production of nitric oxide and TNF. The focus of the current study was to determine if SP-A enhances production of inflammatory mediators by rat macrophages in response to BCG through increased activation of intra-macrophage signalling pathways involving MAP kinases and NFκB. We have examined the role of both the MAPK pathway and NFκB activation in BCG killing and nitric oxide production. We report that both of these pathways are activated by BCG alone and that opsonization of BCG with SP-A leads to enhanced activation of both pathways, contributing to increased intracellular BCG killing.

## Materials and methods

### Materials

[5, 6-^3^H]-Uracil was purchased from NEN (Boston, MA). Fetal bovine serum (FBS) for culture of rat bone marrow macrophages (RBMM) was purchased from HyClone Laboratories; all other tissue culture reagents were from GIBCO-BRL (Grand Island, NY). Kinase assay kits, U0126, and antibodies against phosphorylated and non-phosphorylated ERK1 and ERK2 were obtained from Cell Signalling Technologies (Beverly, MA). All other reagents were purchased from Sigma Chemical (St. Louis, MO).

### Cells and bacteria

Rat bone marrow-derived macrophages (RBMM) were isolated from female Sprague-Dawley rats as previously described [31]. Briefly, femurs were removed from rats and the marrow flushed into 50 ml conical tubes. The cells were resuspended in DMEM and cultured in DMEM with 10% fetal bovine serum (FBS), antibiotics, and 10% L-cell conditioned medium for 5–7 days. Macrophages were then removed from the culture dishes with cold EDTA and plated in 24 or 6 wells dishes as described for each experiment. Prior to infection with BCG, the media was changed to serum- and antibiotic-free DMEM. For NFκB experiments, bone marrow macrophages were prepared from femurs of transgenic mice expressing a luciferase gene driven by the HIV-1 long terminal repeat containing six κB consensus sites in its promoter (obtained from T. Blackwell; [45]).

BCG, Pasteur strain, was obtained from the American Type Culture Collection (Rockville, MD). Bacteria were cultured in Middlebrook Broth (BBL Microbiology Systems) supplemented with OADC enrichment (Laboratory Supply Company, Nashville, TN), and 1.5 ml aliquots of bacteria at approximately 10^8 ^bacteria per ml were stored at -70°C. Colony forming units per ml were determined by plating serial dilutions of the bacteria onto Middlebrook agar plates, and counting colonies after 2–3 weeks of growth.

### Purification of SP-A

SP-A was purified from human alveolar proteinosis fluid (APF) (obtained from Dr. J.R. Wright (Duke University) or Dr. Samuel Hawgood (University of California, San Francisco) as previously described [31]. Briefly, 1–2 ml of APF in PBS was extracted with 25 ml of 1-butanol (Sigma) and then dried overnight under nitrogen. Dried protein was resuspended in 1 mM HEPES buffer, pH 7.5, with 0.15 M NaCl and 20 mM n-octyl-β-D-glucoside. The pellet was collected by centrifugation at 17,000 × g and the process repeated. The final pellet was resuspended in 5 mM HEPES buffer with 1 mM EDTA (pH 7.5) and dialyzed for 48 hours with buffer changes. After dialysis, polymyxin B-agarose was added to the SP-A and the mixture was rotated for one hour at room temperature. The polymyxin B-agarose was removed by centrifugation and the SP-A concentration was determined using the BCA protein kit from Pierce. The final SP-A preparation was divided into 1 ml aliquots and stored at 4°C for immediate use or -20°C for long-term storage. The SP-A was analyzed for purity by SDS-PAGE and for endotoxin contamination using the Limulus amebocyte lysate assay (Associates of Cape Cod, MA). Endotoxin levels were routinely determined to be less than 0.05 units/ml.

### Infections

Frozen stocks of BCG were thawed and vortexed vigorously with a glass bead to break up any clumps. The mycobacteria were collected by centrifugation, and then resuspended in PBS. SP-A or buffer was added, and the mixture incubated for 30 minutes at 37°C. The cells in DMEM were then infected with the opsonized or buffered mycobacteria for the time periods and at the MOIs as indicated in each experiment.

### BCG killing assays

To determine the effect of protein tyrosine kinase inhibitors on BCG killing, a modification of the method of Chan et al. [46] using metabolic labelling of viable BCG was used as follows: cells were incubated with BCG or SP-A-BCG for 4 hr at 37°C. The cells were washed, and DMEM containing 10% serum plus 2.5 μCi of ^3^H-uracil was added to each well. Assays were performed in quadruplicate. At various times from 1 to 5 days, the macrophage monolayers were dissolved in 0.25% SDS and the labelled BCG were collected on GF/C filters, washed extensively with water, dried, and counted in a liquid scintillation counter.

### Nitric oxide assays

Cells were incubated for 24 hr with PBS, SP-A, BCG, or SP-A-BCG in DMEM without serum. Aliquots (100 μl) of the spent media were incubated with an equal volume of freshly prepared Griess reagent (0.5% sulfanilamide and 0.05% naphthylethylenediamidedihydrochloride in 2.5% H_3_PO_4_) for 5 min at room temperature. The level of nitrite as a measure of nitric oxide production was determined spectrophotometrically at 540 nm and compared to standards of sodium nitrite.

### Immunoblot analysis

Cells were incubated with PBS, SP-A, BCG, or SP-A-BCG complexes for 24 hr in serum- and antibiotic-free medium at a ratio of 1:1 BCG:macrophage and 20 μg of SP-A per 5 × 10^5 ^BCG. The cells were washed, and then lysed in immunoprecipitation buffer (20 mM Tris, pH 7.75, containing 1% Triton X-100, 0.5% deoxycholate, 0.15 M NaCl, 0.02% sodium azide, and 0.34 trypsin inhibitory units of aprotinin/ml). Protein concentration in the cell lysate was measured using the BCA protein kit from Pierce, and equal amounts of protein were loaded per lane on a 10% or 4–20% SDS polyacrylamide gel. Proteins were electrophoretically separated, then transferred to nitrocellulose. The nitrocellulose blot was incubated in Tris-buffered saline (TBS) containing either 5% bovine serum albumin (BSA) or 5% milk. The blots were then incubated with the primary antibody indicated in each experiment at the noted concentration. The blot was incubated overnight at 4°C, then washed and incubated with HRP-conjugated goat anti-rabbit IgG (1:10,000). Reactive proteins were visualized by incubation of the blot in 0.2 M Tris-HCl (pH 8.5), 2.5 mM luminol, 0.4 mM *p*-coumaric acid, and 0.0002% H2O2, followed by exposure of X-OMAT film (Kodak, Rochester, NY). In the ERK activation immunoblot experiment, to normalize for protein loading, the blot was stripped with NaOH (200 mM) and reprobed using anti-ERK antibody. Densitometry was performed to quantify protein band intensity using the UN-SCAN-it digitizing system.

### Immunoprecipitation and kinase assays

Cells were incubated with PBS, SP-A, BCG, or SP-A-BCG for varying times as indicated for each experiment. Aliquots (100 μl) of total cell lysate were transferred to microfuge tubes. A 1:25 dilution of antibody directed against the active, phosphorylated form of ERK1/2 was added to each tube and the mixture incubated overnight with rotation at 4°C. Protein A-Sepharose (100 μl) was added to each tube and incubated with rotation at room temperature for 1 hr. Pellets were collected by centrifugation and washed three times with kinase buffer. After the final wash, the pellets were resuspended in kinase buffer and 1 μg of Elk-1-glutathione-S-transferase fusion protein as a substrate in the kinase reaction was added to each tube. The tubes were incubated with rotation at 4°C for 1 hr. SDS-containing sample buffer was added to each tube and samples were resolved by electrophoresis on a 4–20% gradient gel, transferred to nitrocellulose, and analyzed for the presence of phosphorylated substrate by immunoblot with anti-phospho-Elk-1 antibody.

### Electrophoretic mobility shift assays (EMSA)

Cells were incubated with LPS (100 μg), SP-A, BCG, or SP-A-BCG for 30 min. Nuclear extracts were isolated from cells as follows: cells were suspended in lysis buffer (10 mM HEPES, pH 7.9; 10 mM KCl; 0.1 mM EDTA; 0.1 mM EGTA; 0.4% Nonidet P-40; 1 mM dithiothreitol (DTT); 0.5 mM phenylmethylsulfonyl fluoride; and 100 μl protein inhibitor solution (Sigma)), and placed on ice for 10 min. After centrifugation for one minute at 13,000 × g, the nuclei-containing pellet was washed once in lysis buffer, and then suspended in extraction buffer (20 mM HEPES, pH 7.9; 0.4 M NaCl; 1 mM EDTA; 1 mM EGTA; 1 mM DTT; and 100 μl protease inhibitor solution) and vortexed for 15 min at 4°C. Gel shift oligonucleotides containing an NFκB consensus site from the human iNOS promoter (AGTTGAGGGGACTTTCCCAGGC) [47] were end-labelled using T4 polynucleotide kinase (Promega) and [γ-^32^P] ATP. Labelled oligonucleotide (2 × 10^5 ^cmp), single-stranded salmon sperm DNA (200 ng), nuclear extract proteins (10 μg), and binding buffer (20 mM Tris-HCl, pH 7.5; 20% glycerol; 5 mM MgCl_2_; 2.5 mM EDTA; 2.5 mM DTT; 250 mM NaCl; 0.25 mg/ml poly(dI-dC)) were incubated at room temperature for 20 min. A 10-fold excess of unlabeled oligonucleotide was used in the competition assays. Samples were resolved by electrophoresis on 5% polyacrylamide non-denaturing gels in 0.5× Tris-borate-EDTA (TBE) buffer at 150 volts constant. The gels were dried and bands visualized by autoradiography.

### Statistical analyses

The differences between groups were tested using one-way ANOVA. In all cases, a *p *value of < 0.05 was considered significant. Data in figures are expressed as mean ± SD.

## Results

### Herbimycin A inhibits nitric oxide production induced by BCG and SP-A-BCG complexes

**Figure 1:**
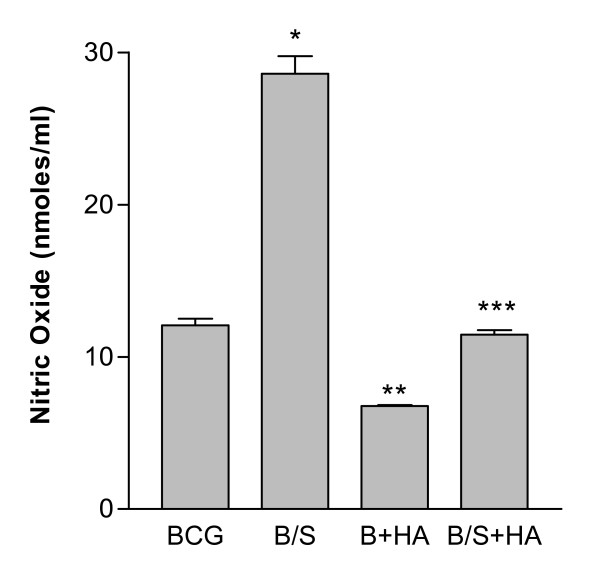
**Herbimycin A inhibits BCG- and SP-A-BCG-induced production of nitric oxide**. BCG were collected by centrifugation, and then suspended in PBS. SP-A (20 μg/5 × 10^5 ^BCG) or buffer was added, and the mixtures incubated for 30 min at 37°C. The BCG (B) or SP-A-BCG (B/S) complexes were pelleted, resuspended in medium, and added to RBMM (5 × 10^5^) in 24 well plates at an MOI of 1. One-half of the cells from each treatment (BCG or SP-A-BCG) were exposed to herbimycin A (HA) at a concentration of 100 nM. Cells plus mycobacteria were incubated for 24 hr in serum-free DMEM. The spent culture medium was removed at 24 hr, and nitrate/nitrite levels were measured using the Griess reagent. Results are the average ± S.D. for triplicate determinations, and are representative of four separate experiments. *p < .001 for B/S compared to BCG; **p < .001 for B+HA compared to BCG; ***p < .001 for B/S + HA compared to B/S.

Activation of intracellular protein tyrosine kinases is a common pathway involved in signalling induced by a variety of pathogens and pathogen-derived products. To determine if BCG-induced production of nitric oxide by rat macrophages in the presence and absence of SP-A involves tyrosine kinase activation, RBMM were incubated with BCG or SP-A-BCG complexes in the presence and absence of 100 nM herbimycin A. As shown in Figure 1, nitrite/nitrate levels in the supernatant of cells treated with BCG alone for 24 hr were approximately 12 nmol/ml. This level was increased 2.5-fold when the BCG was opsonized with SP-A, similar to results previously reported [44]. When cells were pre-incubated with herbimycin A for 30 min prior to infection, nitric oxide production in response to BCG or SPA-BCG complexes was reduced by 60%, suggesting that protein tyrosine phosphorylation is involved in production of nitric oxide in response to BCG or SP-A-BCG complexes. No effect was seen with SP-A or PBS alone.

#### Herbimycin A blocks SP-A-enhanced BCG killing

We have previously reported that SP-A enhances the killing of BCG by rat macrophages. To determine if intracellular growth of BCG is dependent on protein tyrosine phosphorylation, cells were pre-treated with 100 nM herbimycin A for 30 min, then infected with BCG or SP-A-BCG complexes for 4 hr. The cells were washed, and ingested BCG was metabolically labelled with ^3^H-uracil. After incubation for 5 days, the labelled BCG were collected and the associated radioactivity was quantified. The ^3^H-uracil assay is useful in this instance since unlike mammalian host cells the parasite (BCG) can utilize the uracil directly for pyrimidine salvage. ^3^H-Uracil is therefore a valuable counting assay because it allows for pathogen-specific labelling. There should be very little if any labelling of co-purified cellular components. For example, previous studies by Somogyi and Foldes showed that mycobacteria incorporate 80% of ^3^H-uracil into RNA and 20% into DNA [48]. In studies by Aston et al. it was shown that uninfected phagocytes incorporated less than 1% of the ^3^H-uracil used in the experiment [49].

**Figure 2:**
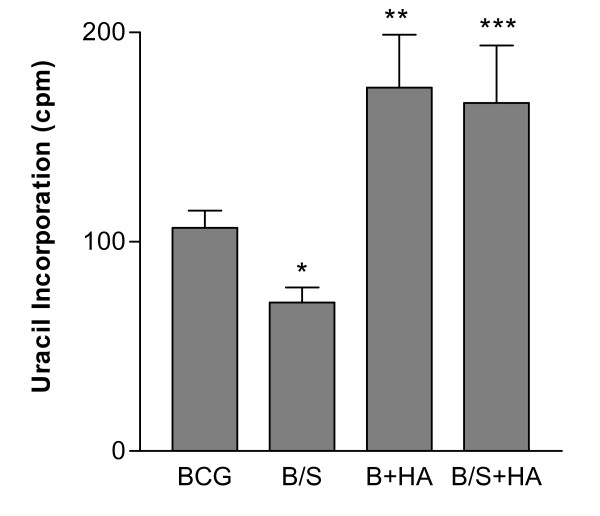
**Herbimycin A inhibits BCG- and SP-A-BCG killing by rat bone marrow macrophages**. RBMM were incubated with BCG or SP-A-BCG (B/S) complexes as described in Figure 1. After removal of unbound BCG, cells plus ingested organisms were supplied with fresh medium minus antibiotics, plus serum containing 2 μCi per well of ^3^H-uracil. After five days incubation, macrophages were lysed with SDS, and viable BCG were collected by filtration over GF/C filters. The filters were dried, and then counted by liquid scintillation counting. Viability of macrophages in companion wells was verified by vital dye exclusion. Results shown are the average of quadruplicate determinations ± S.D., and are representative of two separate experiments. * = p < .001 for BCG compared to SP-A/BCG; ** = p < .001 for SP-A/BCG + NMMA compared to BCG and SP-A/BCG.

As shown in Figure 2, SP-A reduced the level of intracellular BCG growth by approximately 40%, in agreement with previous reports [44]. Inclusion of herbimycin A blocked intra-macrophage BCG killing, both in the presence and absence of SP-A, as evidenced by the increase in labelled BCG. These results suggest that tyrosine kinases are involved in induction of nitric oxide and subsequent BCG killing, both in the presence and absence of SP-A. Qualitative determination of cell survival in the presence or absence of herbimycin A was performed by trypan blue exclusion. After five days, there was no evidence of a decrease in cell viability.

### SP-A enhances ERK1/2 activation in the presence of BCG

**Figure 3:**
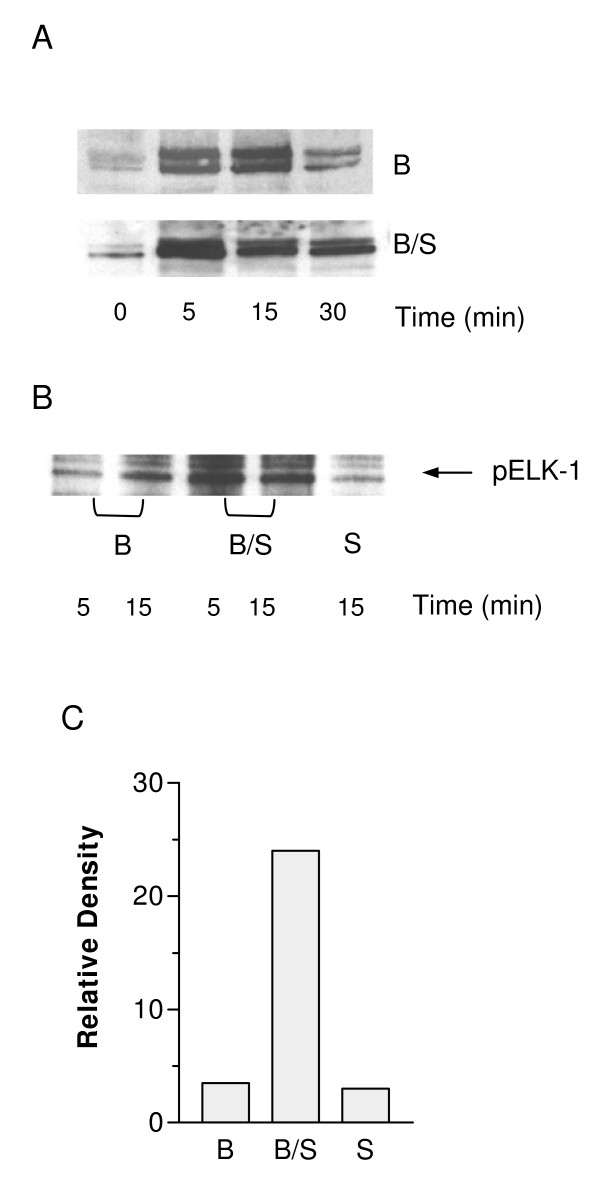
**SP-A enhances BCG-induced ERK1/2 MAP kinase activation**. Panel A: RBMM were incubated with BCG or SP-A-BCG complexes as described in Figure 1 for 0–30 min. At each time point, cells were washed with cold PBS containing 100 μM sodium vanadate to remove any uningested BCG and to inactivate phosphatase activity. Cells were solubilized in immunoprecipitation buffer and total proteins were isolated. Extracts were analyzed by SDS-PAGE, followed by transfer to nitrocellulose, and analysis by Western blot using an antibody specific for phosphorylated forms or ERK-1 and ERK-2. Panel B: RBMM were incubated for the indicated times with BCG, SP-A-BCG, or SP-A alone. Total protein was extracted as described above. Activated ERK-1/2 was immunoprecipitated using a phospho-specific antibody. The antibody-ERK-1/2 complex was then added to a mixture containing ATP and a GST-Elk-1 fusion protein and allowed to incubate for 5 min. The proteins were separated by SDS-PAGE and phosphorylated Elk-1 was visualized by Western blot analysis. Panel C: bands from the blots shown in panel B corresponding to phosphorylated Elk-1 after 5 min treatment with immunoprecipitated ERK-1/2 were quantified using image analysis. Blots are representative of three independent experiments and were normalized for equal protein loading by Western blot analysis for non-phosphorylated proteins within the same membrane.

Several groups have identified MAP kinase family members as key targets of PTKs and participants in signalling cascades leading to the induction of proinflammatory mediators. To determine if two of these family members, ERK-1 and ERK-2, are involved in BCG and SP-A-BCG signalling, immunoblot analysis was used to examine the level of ERK phosphorylation as a measure of ERK activation. Cells were incubated for the indicated times with BCG or SP-A-BCG. At each time point, cells were washed, and then solubilized in immunoprecipitation buffer. Extracts were analyzed by immunoblot analysis, using an antibody specific for the phosphorylated forms of ERK-1 and ERK-2. As shown in Figure 3A, in cells stimulated with BCG alone, both ERK-1 and ERK-2 were phosphorylated. ERK phosphorylation was observed to be minimal in cells incubated in medium (data not shown) or SP-A alone which was found to be roughly equivalent to levels seen with BCG alone (Figure 3C). Maximal stimulation appeared at 15 min, followed by diminution of the signal at 30 min. In cells treated with SP-A-BCG, a stronger signal was evident at 5 min, and the phosphorylation was sustained through 30 min.

To determine if the enhanced phosphorylation of ERK-1 and ERK-2 correlated with increased kinase activity, in vitro kinase assays were performed. Cells were treated with BCG or SPA-BCG for 5 and 15 min. Control cells were incubated for 15 min with SP-A alone. Total cellular protein was extracted, and phosphorylated ERK-1/2 was immunoprecipitated using a polyclonal antibody specific for the phosphorylated forms of both enzymes. The immunoprecipitates were then incubated with kinase buffer and Elk-1-glutathione-S-transferase fusion protein as a substrate in the kinase reaction. ERK activation was then determined by immunoblot analysis of the cell extracts using anti-phospho-Elk-1 antibody. As shown in Figure 3B, treatment of RBMM with BCG for 5 or 15 min resulted in increased phosphorylation of the Elk-1 substrate compared to SP-A alone, and this activation was significantly increased by opsonization of the BCG with SP-A. Figure 3C, shows densitometric quantitation of the bands from the five-minute treatments of cells with BCG, BCG + SP-A, and SP-A, as well as the positive control of Elk-1 fusion protein incubated with commercially available activated Erk-2 protein. Results demonstrate that there is a significant increase in the phosphorylation of Elk-1 in cells treated with BCG + SP-A versus BCG alone suggesting greater activation of Erk-1/2 in those cells. These results suggest that BCG signalling involves ERK kinases, and that SP-A enhances the activation of this pathway.

### ERK inhibitors block SP-A-enhanced nitric oxide production

**Figure 4:**
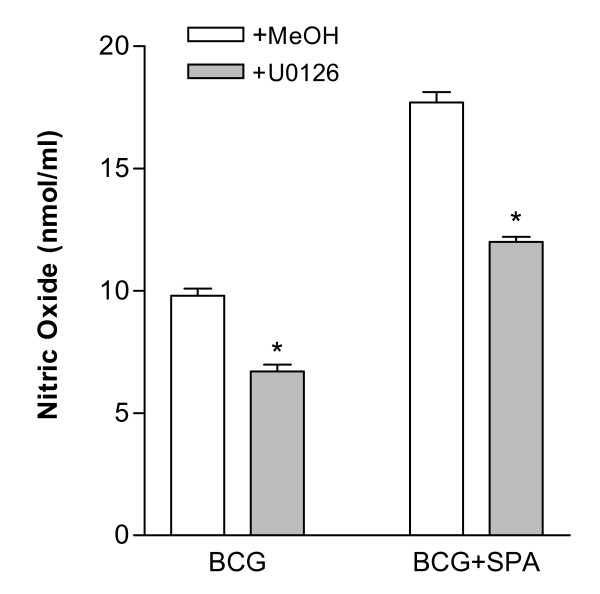
**Inhibition of ERK-1/2 results in decreased nitric oxide levels**. RBMM were pre-treated with U0126 (1 μM) or vehicle (MeOH) for 30 min prior to infection as described in Figure 1. Cell supernatants were analyzed for nitric oxide production after 24 hr. *p < 0.001 for BCG vs BCG+U, BCG+SP-A vs BCG+SP-A+U; n = 3.

To determine if ERK activation in response to BCG resulted in production of nitric oxide, cells were pre-treated with U0126, an inhibitor of the upstream kinases MEK-1 and MEK-2 required for ERK activation. U0126 (1 μM) or methanol (vehicle) was added to RBMM 30 min prior to incubation with PBS, SP-A, BCG, or SP-A-BCG. After 24 hr, nitric oxide levels in the media were measured. As shown in Figure 4, U0126 reduced nitric oxide production in cells treated with either BCG or SP-A-BCG by approximately 35%.

### SP-A enhances the BCG-induced activation of NFkB

**Figure 5:**
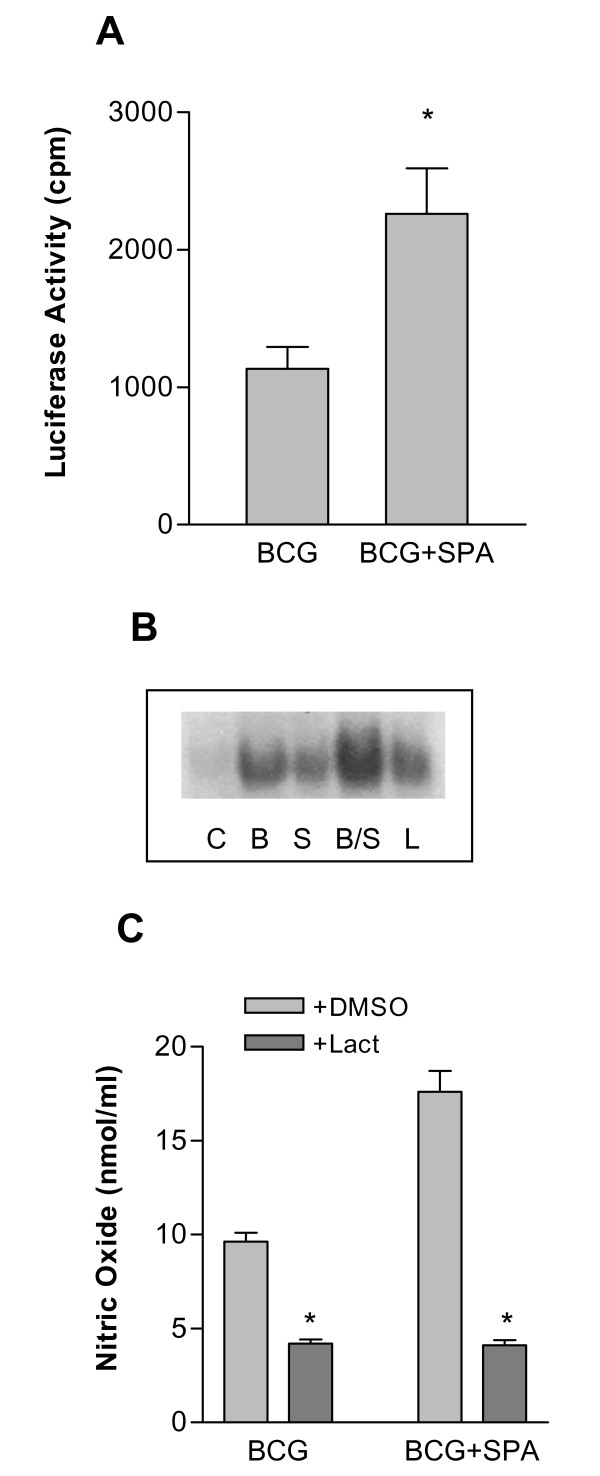
**SP-A enhances BCG-induced NFκB activation**. Panel A: RBMM were obtained from an HIV-1-LTR-luciferase (HLL) transgenic mouse. Mature macrophages were infected for 24 hr with BCG or SP-A-BCG as described in Figure 1. Cells were lysed and luciferase activity was detected by luminometry. Relative light units were corrected by total protein content. *p < 0.05, n = 3. Panel B: RBMM were infected with BCG, SP-A, or SP-A-BCG as described in Figure 1 for 30 min. Nuclear proteins were extracted as described in Methods, and incubated with a ^32^P-labeled oligonucleotide containing a consensus NFκB binding sequence. Protein-oligonucleotide complexes were then resolved by electrophoresis in a non-reducing polyacrylamide gel. The gel was dried and exposed to film for visualization of bands. LPS at a concentration of 1 μg/ml was run as a positive control (L). Panel C: RBMM were incubated with 1 mM lactacystin for 30 min prior to infection with BCG or SP-A-BCG as described in Figure 1. Nitric oxide was measured in the supernatant after 24 hr. * = p < 0.05, n = 3.

Several groups have recently reported activation of NFκB in response to both intact mycobacteria and mycobacterial cell wall components [25-27]. To determine if BCG infection of rat macrophages leads to activation of NFkB, two separate strategies were used. First, macrophages from mice engineered to constitutively express a luciferase reporter gene driven by a kB-containing promoter were incubated with BCG or SP-A-BCG complexes. After 24 hr, luciferase activity was measured. As shown in Figure 5A, SP-A enhanced the BCG-induced activation of the NFκB promoter by approximately 2-fold. This was further confirmed by gel shift analysis as shown in Figure 5B. Little or no effect was seen with SP-A alone. To determine if NFκB activation plays a role in BCG- and SP-A-BCG-induced nitric oxide production, RBMM were incubated with lactacystin which blocks NFκB activation by preventing IκB degradation and release from the NFκB complex [50]. Cells were pre-incubated with lactacystin or vehicle (DMSO) for 30 min, then BCG or SP-A-BCG were added for an additional 24 hr. Nitric oxide was measured in the supernatant as nitrate/nitrite. As shown in Figure 5C, SP-A enhanced the production of nitric oxide, in agreement with previous results [42], and lactacystin completely blocked this effect suggesting that NFκB activation plays an important role in BCG- and SP-A-BCG-induced nitric oxide release.

## Discussion

Mycobacteria are obligate intra-macrophage organisms, and must devise ways to avoid triggering the host response leading to microbe killing. It is therefore likely that interaction of virulent mycobacteria with host macrophages will lead to minimal production of inflammatory mediators and limited activation of anti-microbial processes. In previous studies we have shown that SP-A enhances BCG-induced production of nitric oxide and TNF, resulting in increased BCG killing by the infected macrophages [44]. A common signaling pathway leading to activation of the iNOS gene is phosphorylation of cellular targets, mediated in part by the MAP kinase family. In addition, binding of the transcription factor NFκB to the iNOS promoter is known to be involved in nitric oxide production. In the current study we have focused our attention on the role that SP-A plays in enhancing signaling in macrophages infected with BCG. Specifically we have examined the effect of SP-A on activation of the MAP kinases ERK1/2 and the transcription factor NFκB.

In initial experiments we found that a general inhibitor of PTKs (herbimycin A) blocked both the BCG- and SP-A-BCG-induced production of nitric oxide and the killing of internalized BCG, suggesting that one or more cellular kinases was required for signalling. An important downstream target of cellular PTKs is the family of MAP kinases that are activated following phosphorylation. These serine/threonine kinases then phosphorylate and activate downstream targets such as specific transcription factors that lead to modulation of gene expression. In the current study we found that BCG alone activated ERK1/2 with maximal stimulation at 15 min. SP-A enhanced and prolonged this activation with a maximal effect at 5 min. Inhibitors of upstream kinases blocked nitric oxide production in the presence of both BCG and SP-A-BCG, further supporting a role for this pathway during BCG infection. These results suggest that the ability of SP-A to enhance BCG killing as previously described involves activation of the MAP kinases ERK1/2.

These studies are supported by work from other laboratories demonstrating a role of members of the MAP kinase family in mycobacterial signalling, but the specific members of the family that play a role appear to be dependent on the mycobacterial species as well as the source and functional status of the macrophages used for study. For example, Reiling et al. reported that *M. avium*-induced TNF production in human monocyte-derived macrophages involved ERK but not p38 [20]. Blumenthal et al reported that interaction of *M. avium *with mouse bone marrow macrophages resulted in TNF production that was dependent on ERK activation but did not involve stimulation of p38 [51]. In contrast, Tse reported that all three kinases – p38, ERK, and JNK – were involved in *M. avium*-induced TNF production in mouse bone marrow macrophages [52], and Roach and Schorey showed that virulent *M. avium *activated ERK and p38 but not JNK in the same cells [8]. Chan reported that the LAM from *M. tuberculosis *activated ERK and JNK but not p38 in RAW cells [18]. We have preliminary data showing that p38 and JNK are not activated to any significant level following BCG or SP-A-BCG infection of rat macrophages (data not shown).

There is a growing body of evidence that survival of intra-macrophage pathogens is linked to activation and deactivation of intracellular kinases. Studies with *Leishmania *have shown that entry of organisms into non-activated macrophages is accompanied by activation of protein tyrosine phosphatases that inactivate MAP kinases through removal of phosphate groups [53]. When *Leishmania *organisms are internalized by stimulated macrophages, MAP kinases are activated with concomitant production of proinflammatory mediators. Ibata-Ombetta reported that *Candida *was able to prolong survival in macrophages by specific activation of MAP kinase phosphatase (MKP)-1, leading to deactivation of ERK1/2 [21]. Henning et al. also recently reported that SP-A can decrease the phosphorylation of Akt potentially affecting MAP kinases and NF-κB [54]. Thus, a key strategy for these pathogens in evading intra-macrophage killing might involve regulation of MAP kinases leading to enhanced production of inflammatory mediators. We have preliminary data showing that BCG alone activates the phosphatase SHP-2, and pre-incubation of the BCG with SP-A attenuates this activation, suggesting that SP-A might enhance BCG killing through alteration of the kinase-phosphatase balance.

It has been suggested that the MAP kinase-mediated increase in the production of inflammatory mediators may involve activation of transcription factors such as NFκB, although a direct link leading from MAP kinase activation to NFκB activation has not been established. In the current study we have shown that BCG and SP-A-BCG complexes activate NFκB in addition to members of the MAP kinase family, but we cannot definitely say that NFκB activation is dependent on MAP kinase activity. Manucso et al. reported that the NFκB inhibitor CAPE blocked GBS-stimulated TNF production, however ERK inhibitors did not alter p50/p65 activation, suggesting two independent pathways [15]. Carter et al. reported that p38 regulates NFkB-dependent gene transcription by activating TFIID, but inhibitors of p38 did not alter NFkB activation, again suggesting that these two pathways are independent [55].

Receptors that might be involved in mediating mycobacterial or SP-A-mycobacterial effects are not yet known. The mycobacteria species that have some clinical relevance – including *M. tuberculosis, M. avium*, and BCG – all have high mannose groups exposed on their surfaces, making them good candidates for mannose receptor ligands [56]. In support of this, Schlesinger and co-workers reported that *M. tuberculosis *was internalized by human monocyte-derived macrophages through the mannose receptor in the absence of opsonins. However, there is no report directly linking mycobacterial binding to the mannose receptor to activation of signalling pathways. In fact, Reiling et al. reported that *M. avium*-induced TNF production by human monocyte-derived macrophages was blocked by anti-CD14 antibodies but not my anti-mannose receptor antibodies [20]. More recent studies using mycobacterial components have suggested that mycobacteria might interact with toll-like receptors (TLRs) on the macrophage surface [26,27,57,58]. We have suggested previously that SP-A redirects mycobacteria to interact with the SP-A-specific receptor SPR210 [31,59]. Anti-SPR210 antibodies block SP-A binding, inhibit ingestion of SP-A-BCG complexes, and reduce SP-A-BCG-mediated production of nitric oxide. The molecular characterization of this receptor is currently underway, and no information is yet known about specific interaction of the SPR210 with components of the intracellular signalling pathways.

In the current and previous studies we have found no effect of SP-A alone on RBMM function. Only when attached to a particulate material does SP-A appear to induce signalling in RBMM leading to production of inflammatory mediators. This is somewhat controversial, since other groups have found that SP-A alone has an effect on resident macrophages. For example, early studies from several laboratories reported that SP-A interaction with macrophages and macrophage cell lines resulted in production of reactive oxygen and nitrogen species and inflammatory cytokines, and activated NFκB [60-64]. Vazquez et al. recently reported that SP-A induced the expression of matrix metalloproteinase (MMP)-9 in human MDM, and this activation appeared to involve TLR2 [65]. Murakami et al. reported that a direct interaction of SP-A with TLR2 on U937 macrophages altered peptidoglycan-induced cell signalling [58]. Most likely the specific SP-A preparations used and the source of the macrophages affect these findings, and careful examination of need to sort out these differences to fully define the role of SP-A in innate host defense.

Although we have shown that SP-A enhances killing of BCG by rat macrophages, this does not appear to be the case with *M. avium*. In previous work we have shown that SP-A increases *M. avium *ingestion by RBMM and enhances production of both TNF and nitric oxide [44]. However, SP-A had no effect on intra-macrophage survival of the ingested *M. avium*. Gomes et al. reported that *M. avium *growth was enhanced in the presence of nitric oxide [66], and Tse et al. reported that inhibition of MAP kinase inhibited *M. avium *growth [48]. One might predict therefore that SP-A would enhance the activation of the MAP kinase signalling pathway by *M. avium*, leading to continued and possibly enhanced intracellular growth. The effect of SP-A on pathogen survival may be directly linked to the specific signalling pathways turned on by each pathogen, and SP-A may not be able to overcome alternative cellular pathways activated by certain pathogens.

## Conclusion

This is the first report demonstrating that SP-A increases mediator production in response to mycobacteria through activation of MAP kinases and NFκB. Like other intra-macrophage pathogens, mycobacteria have evolved a variety of strategies for evading host defense, including limitation of the ability of the host cell to trigger important signalling pathways. In the lung, during the first insult by mycobacteria, SP-A may play a role in the response of uninfected, non-activated alveolar macrophages by enhancing their capacity to activate signalling pathways, thus turning on necessary defense genes such as iNOS and TNF. The role of SP-A is complex, and may depend directly on the nature of the pathogen and the state of activation of the macrophages. In addition, SP-A may interact differently with mycobacteria released from macrophages as opposed to mycobacteria in the initial onslaught. These questions are currently being addressed in our laboratory.

## Abbreviations

(BCG): bacillus Calmette-Guerin; (PTK): protein tyrosine kinase; (ERK): extracellular signal regulated kinase; (MAP kinase): mitogen-activated protein kinase; (JNK): c-Jun amino terminal kinase; (LPS): lipopolysaccharide; (LAM): lipoarabinomannan; (NFκB): nuclear factor κB; (SP-A): surfactant protein A; (RBMM): rat bone marrow macrophages; (FBS): fetal bovine serum; (TLR): toll-like receptor.

## Competing interests

The authors declare that they have no competing interests.

## Authors' contributions

JL carried out the immunoblot analyses, the inhibitor studies, the NFkB assays, and the enzymatic assays. DV participated in the design and coordination of the study, and helped to draft the manuscript. VS conducted the killing assays, conceived of the study, participated in the design, and supervised the experimental work. All authors read and approved the final manuscript.
